# Mechanotactile Sensory Feedback Improves Embodiment of a Prosthetic Hand During Active Use

**DOI:** 10.3389/fnins.2020.00263

**Published:** 2020-03-26

**Authors:** Ahmed W. Shehata, Mayank Rehani, Zaheera E. Jassat, Jacqueline S. Hebert

**Affiliations:** ^1^Division of Physical Medicine and Rehabilitation, Department of Medicine, Faculty of Medicine and Dentistry, University of Alberta, Edmonton, AB, Canada; ^2^Glenrose Rehabilitation Hospital, Alberta Health Services, Edmonton, AB, Canada

**Keywords:** rubber hand illusion, prosthetics, sensory feedback, embodiment, motor learning, electromyography, simulated prosthesis

## Abstract

There have been several advancements in the field of myoelectric prostheses to improve dexterity and restore hand grasp patterns for persons with upper limb loss, including robust control strategies, novel sensory feedback, and multifunction prosthetic terminal devices. Although these advancements have shown to improve prosthesis performance, a key element that may further improve acceptance is often overlooked. Embodiment, which encompasses the feeling of owning, controlling and locating the device without the need to constantly look at it, has been shown to be affected by sensory feedback. However, the specific aspects of embodiment that are influenced are not clearly understood, particularly when a prosthesis is actively controlled. In this work, we used a sensorized simulated prosthesis in able-bodied participants to investigate the contribution of sensory feedback, active motor control, and the combination of both to the components of embodiment; using a common methodology in the literature, namely the rubber hand illusion (RHI). Our results indicate that (1) the sensorized simulated prosthesis may be embodied by able-bodied users in a similar fashion as prosthetic devices embodied by persons with upper limb amputation, and (2) mechanotactile sensory feedback might not only be useful for improving certain aspects of embodiment, i.e., ownership and location, but also may have a modulating effect on other aspects, namely sense of agency, when provided asynchronously during active motor control tasks. This work may allow us to further investigate and manipulate factors contributing to the complex phenomenon of embodiment in relation to active motor control of a device, enabling future study of more precise quantitative measures of embodiment that do not rely as much on subjective perception.

## Introduction

Persons with upper limb amputation face significant limitations in performing activities of daily living. Myoelectric prosthesis, controlled by electrical signals extracted from residual limb muscles, provide a potentially feasible solution (Belter et al., 2013; van der Riet et al., 2013; Geethanjali, 2016), however, dissatisfaction and rejection of the prosthesis remains high, with some studies reporting up to 75% of myoelectric prosthesis users abandoning their device (Biddiss and Chau, 2007). Although there have been several advancements to improve dexterity and restore hand grasp patterns (Gallagher, 1986; Murray, 2004; Giummarra et al., 2008), myoelectric prostheses do not provide continuous feedback to allow real-time regulation of muscle contraction. The lack of feedback poses a significant challenge to the prosthesis user; without such sensory feedback, the prosthesis needs near-constant visual attention and mental concentration to operate (Sobuh et al., 2014; Hebert et al., 2019).

Sensory feedback has thus been highlighted as a possible missing element for improving the acceptance of upper limb prosthetic devices (Ehrsson et al., 2004; Longo et al., 2008). One hypothesis is that sensory feedback will restore the feeling of ownership of the prosthesis as part of the body, by facilitating integration of the prosthesis into the body representation (Gallagher, 1986; Murray, 2004). Although ownership can be induced by providing sensory input matched to natural sensation, i.e., pressure proportionally matching touch sensation, in an expected location and orientation (Giummarra et al., 2008), embodiment is likely a more complex phenomenon. Embodiment is thought to involve sub-components of ownership (the feeling that the hand is actually a part of the body), location (the sensation that the hand is in an appropriate area and that a relationship exists between what is seen in that area and where it is felt on the hand) and agency (a feeling of control over the actions of the hand) (Ehrsson et al., 2004; Longo et al., 2008). These items interrelate and may result in a foreign object, such as the prosthetic hand, being integrated into the body schema (Gallagher and Cole, 1995), which may increase acceptance and use of the prosthesis.

Prior research has used an experimental paradigm called the rubber hand illusion (RHI) to elicit the sense of embodiment by applying synchronous stimulation to a rubber hand and the participant’s hand, demonstrating that the sense of body ownership is closely associated with cutaneous touch (Botvinick and Cohen, 1998; Ehrsson et al., 2004; Tsakiris and Haggard, 2005; Longo et al., 2008). The RHI is a robust phenomenon and appears to be sensitive to the relative strength of the tactile input (Ehrsson et al., 2008). Tactile input has been shown to induce this illusion even if it is modality mismatched (D’Alonzo and Cipriani, 2012), however, the input needs to be delivered synchronously in order to preserve the illusion (Armel and Ramachandran, 2003; Ehrsson et al., 2004; D’Alonzo and Cipriani, 2012). In persons with upper limb amputation who have undergone the targeted reinnervation procedure, the provision of direct cutaneous touch feedback to the residual limb has been reported to create a vivid illusion of ownership of a passive prosthetic hand (Marasco et al., 2011), similar to other populations with upper limb amputation when synchronous touches were applied to their residual limb and a rubber hand (Ehrsson et al., 2008) or a robotic hand (Rosen et al., 2009).

Adding active control of the hand has been shown to enhance the experience of the RHI; both able-bodied and participants with amputation have been shown to experience a sense of ownership over the robotic hand when they were remotely controlling the robotic movements (Rosen et al., 2009; Sato et al., 2018). In fact, active motor control of a congruent movement was shown to induce both ownership and agency, without a significant effect of additional brushing feedback (Sato et al., 2018). Studies in participants with amputation have further shown that embodiment responses can be positive with motor control alone as well as with sensory feedback provided by peripheral intraneural stimulation (Graczyk et al., 2018; Page et al., 2018), and that the naturalness of the tactile sensation elicited by nerve stimulation may affect embodiment responses (Valle et al., 2018). Furthermore, in other participants with wearable closed-loop control prosthetic systems, it has been shown that kinesthetic feedback enhances agency but not ownership (Marasco et al., 2018).

There is, therefore, building evidence that multisensory inputs of both sensation and motoric cues can enhance the sense of ownership (Kalckert and Ehrsson, 2014). However, there remains some inconsistency in the literature regarding the relative contribution of active motor control with or without concordant sensory stimulation to agency and ownership. The ability to further investigate these factors is limited by the small number of participants with bidirectional sensory feedback systems and the limitations of altering their sensory feedback parameters to explore the impact of feedback type.

A common technique used to study myoelectric prosthesis function is the use of simulated devices on able-bodied research participants as an approximation to prostheses used by persons with upper-limb amputation (Panarese et al., 2009; Amsuess et al., 2016; Johansen et al., 2016; Clemente et al., 2017; White et al., 2017). We designed such a device to allow the manipulation of sensory feedback during motor control (Kuus et al., 2017), to investigate the factors of ownership, location, and agency in a wearable prosthesis. The objective of the current work was to assess the embodiment responses of participants using a wearable simulated prosthesis platform providing mechanotactile feedback during active motor control. In order to ascertain the validity of this approach in comparison to the classic RHI, we first had to determine the influence of type of feedback (mechanotactile tapping versus brushing) in a passive condition with the worn prosthesis simulator (the “passive prosthesis test”), followed by investigation of the contributions of active motor control to the embodiment phenomenon (the “active prosthesis test”).

## Materials and Methods

In this study, we used a simulated prosthesis that allows the integration of sensors and mechanotactile feedback tactors (Kuus et al., 2017). The study was divided into two test phases – passive prosthesis test and active prosthesis test.

### Study Participants

Twenty-one able-bodied individuals were recruited to participate in this study [12 females; age: 31.9 ± 9.3 (mean ± SD); 3 left-hand dominant]. All participants were over 18 years old with no upper limb dysfunction (no muscular or neurological dysfunction, no sensory deficit in the hand), normal or corrected to normal vision, and no upper limb surgery in the past year. Only 1 participant had previous experience operating a simulated prosthesis. Written informed consent according to the University of Alberta Health Research Ethics Board was obtained from all participants before conducting the experiment (Pro00057340).

### Experimental Setup

Participants sat comfortably on a chair in front of a table and facing the experimenter. The height of the chair was adjusted to ensure that the participant’s arm was resting on the table. A black sheet was placed over the participant’s shoulder to ensure that their arm was completely obscured. Noise-canceling headphones were placed over the participant’s ears during testing but were removed during instruction phases and setup.

#### Device Setup and Control Parameters

The participant’s right arm was secured in an adjustable brace that comfortably restricted wrist movement. A prosthetic hand was secured to the brace such that it was oriented beneath the participant’s right hand, similar to the specifications outlined by Kuus et al. (2017) with adjustments of the strapping to allow access to the participant’s hand. The prosthetic hand was controlled using two-cite proportional myoelectric control (Battye et al., 1955) with one of the electromyography (EMG) sensors placed on the wrist extensor muscle and the other placed on the wrist flexor muscle. Muscle contractions at these sites were mapped to the velocity of the opening and closing of the single degree of freedom prosthetic hand. EMG sensor gains were adjusted to ensure easy and reliable control of the prosthetic hand. The participant was free to move around with the brace attached during training to use the device, but the testing occurred in a seated position resting the device on the table.

#### Tactor Setup

Three mechanotactile tactors integrated into the brace were aligned to stimulate the thumb, index, and middle fingers of the participant to relay tactile feedback to participants. These tactors pushed on the participant’s fingers by converting rotational motion from a motor using rack and pinion gears to linear motion (Figure 6a in Schoepp et al., 2018). The linear displacement of these actuators on the fingertips was mapped proportionally to the force sensed using force-sensitive resistor sensors that were placed on the corresponding thumb, index, and middle fingers of the prosthetic hand (Saunders and Vijayakumar, 2011). This system had an average delay in response of 92 ± 16 ms, which is below the recommended threshold to evoke embodiment (Ismail and Shimada, 2016).

### Experimental Protocol

**FIGURE 1:**
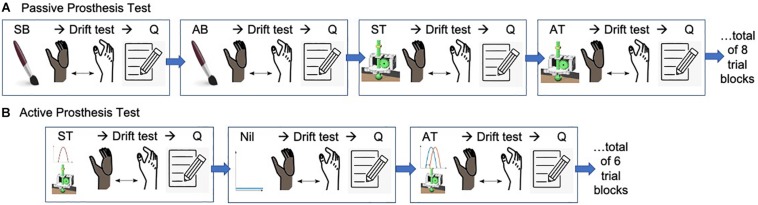
Overview of experimental protocol. **(A)** A representative randomization sequence for the Passive Prosthesis Test. There were 4 feedback conditions tested: Synchronous Brushing (SB), Asynchronous Brushing (AB), Synchronous Tapping (ST), and Asynchronous Tapping (AT). A single trial block consisted of the experimenter applying one of the feedback conditions, followed by the participant performing the drift test, and then filling out the questionnaire (Q). Participants were randomized into one of ten predetermined randomization sequences, each consisting of 2 repetitions of each of the 4 conditions, presented in a random order for a total of 8 trial blocks. **(B)** A representative randomization sequence for the Active Prosthesis Test. For this test, the participant actively controlled the prosthesis in 3 feedback conditions: Synchronous Tapping (ST), Asynchronous Tapping (AT), and no feedback (Nil), for which the tactors were turned off. A trial block consisted of the participant actively using the prosthesis for grasp activities with one of the feedback conditions, followed by an assessment of proprioceptive drift, and then filling out the questionnaire. Participants were randomized to one of ten randomized sequences, consisting of 2 repetitions of each of the 3 conditions, presented in a random order for a total of 6 trial blocks.

In the first portion of this study, we investigated the effect of receiving two types of feedback (brushing and tapping) with and without delay (asynchronous and synchronous, respectively) on the sense of embodiment of the prosthetic hand while wearing the device during a Passive Prosthesis Test. We then investigated the effect of providing no feedback, synchronous tapping feedback, or asynchronous (delayed) tapping feedback during an Active Prosthesis Test. After each test and for each feedback condition, participants were asked to perform an assessment of proprioceptive drift followed by filling out a questionnaire. An overview of the experimental protocol is provided in Figure 1.

#### Passive Prosthesis Test

**FIGURE 2:**
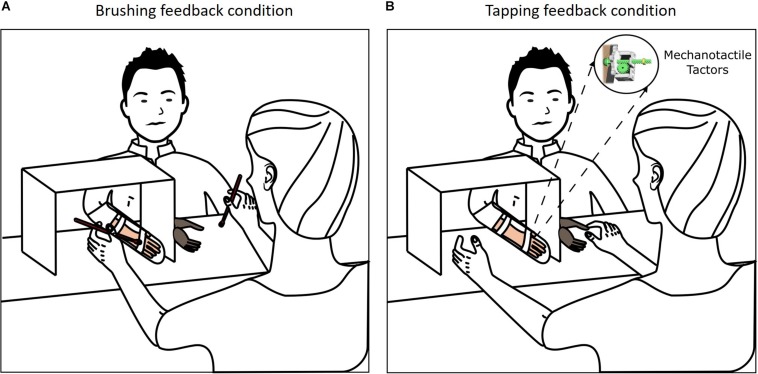
Experimental setup for Passive Prosthesis Test. **(A)** Brushing feedback condition. The brushing stimulation was administered by the experimenter to the prosthetic hand and to the participant’s hand with two paintbrushes. **(B)** Tapping feedback condition. Three mechanotactile tactors placed on the thumb, index and middle fingers of the participant delivered a mechanotactile stimulus when the experimenter applied pressure to the corresponding sensors on the fingertips of the prosthetic hand.

A box that was accessible from both the front and back sides was placed on the table in front of the participant. This box was placed over the participant’s right arm between the brace and the prosthetic hand (Figure 2). The black sheet that was covering the participant’s arm and shoulder was adjusted if needed. In this manner, the participant could see only the prosthetic hand and forearm section, but not their real hand or forearm. The experimenter administered various conditions of feedback stimuli to the participant’s obscured right hand and to the visible prosthetic hand.

The combination of two different feedback types (brushing and tapping) and two different feedback timing (synchronous and asynchronous) yielded four different conditions of feedback stimuli. These conditions were: Synchronous Brushing (SB), Asynchronous Brushing (AB), Synchronous Tapping (ST), and Asynchronous Tapping (AT) (described in Table 1). A single trial block for each condition consisted of the experimenter applying the feedback stimulus, followed by the participant performing an assessment of proprioceptive drift and then filling out a questionnaire (Appendix A).

**TABLE 1:** Feedback stimuli provided to the participant in random order during the passive prosthesis test.

**Feedback stimulus**	**Description**
Synchronous brushing (SB)	Both the prosthetic hand (in view of the participant) and the obscured participant’s right hand were stroked with a paintbrush at the same time, location, and duration. Stimulation was delivered randomly to each location with varying durations (Figure 1A)
Asynchronous brushing (AB)	The prosthetic hand was stroked with a paintbrush at the same location as the participant’s hand, but at a different time and duration. Stimulation was delivered randomly to different locations with varying durations.
Synchronous tapping (ST)	The sensorized fingers of the prosthetic hand were pressed resulting in the mechanotactile tactor applying pressure on the corresponding finger of the participant. Stimulation was delivered randomly to each finger with varying durations and pressures (Figure 1B)
Asynchronous tapping (AT)	A time delay was introduced into the mechanotactile tactor program resulting in a delayed response of 500 ms to pressure applied on the sensorized prosthetic finger. The sensorized fingers of the prosthetic hand were pressed resulting in the mechanotactile tactor applying a 500 ms delayed pressure on the corresponding finger of the participant. Stimulation was delivered randomly to each finger with varying durations and pressures.

Participants were randomized into one of ten predetermined randomization sequences (Figure 1A). Each of these sequences consisted of 2 repetitions of each of the 4 conditions, presented in a random order for a total of 8 trial blocks (2 × 4).

#### Active Prosthesis Test

Participants had active motor control of the prosthetic hand, as per device setup 2.2.1. The participants were provided with a period of functional task training, wherein they used the simulated prosthesis to grasp and move objects with the prosthetic hand in a structured environment. They were encouraged to grasp and release a variety of objects of different sizes and densities (soft balls, blocks, plastic cups) to ensure adequate control and to experience the sensory feedback. Objects were then presented in a predetermined order, and participants were instructed to move the object to different positions or to stack them. Participants were asked to be as precise as possible and told that the time taken for each manipulation was not going to be considered, so that they would focus on the sensory experience and control rather than speed of moving the objects. Once they completed the object manipulation sequence, the participant would immediately rest their arm and device on the table.

For this test, three feedback conditions were tested, namely ST, AT, and no feedback. For all conditions, the participants had the same active motor control of the prosthetic hand. A trial block consisted of the participant actively using the prosthesis for grasp activities with one of the feedback conditions, followed by an assessment of proprioceptive drift, and then filling out the questionnaire. Participants were randomized to one of ten randomized sequences (Figure 1B). Each of these sequences consisted of two repetitions of each of the six conditions, presented in a random order for a total of six trial blocks.

During ST condition block, participants felt the mechanotactile tactor push on their right-hand thumb, index, and middle fingers when using the prosthetic hand to grasp objects. Ismail and Shimada (Ismail and Shimada, 2016) showed that participants felt significant weaker sense of agency with temporal delays of 240–490 ms; therefore, for the AT condition block, the mechanotactile tactors were delayed by 500 ms, and for the no feedback condition the tactors were switched off. For all conditions, the participants had the same active motor control of the prosthetic hand.

### Outcome Measures

Following each testing condition, participants were asked to (a) with vision obscured, point with their left index finger on the board where they thought the tip of their actual index finger was (to measure proprioceptive drift) and (b) rate their agreement with 10 questions in the embodiment survey using a visual analog scale, adapted from previous work in this area (Ehrsson et al., 2008; Longo et al., 2008).

#### Proprioceptive Drift

The proprioceptive drift, outlined by Tsakiris et al. (2005), was calculated by measuring the difference between the points at which the participant indicated the position of their index finger pre- and post-test. With eyes closed, participants were instructed to point with their left index finger where they thought the tip of their finger was before and after a test. A positive result (positive drift) was indicative of the participant locating their hand toward the prosthetic hand. A negative result (negative drift) indicated that the participant had located their hand further away from the prosthetic hand.

#### Embodiment Questionnaire

Ten questions (five control and five related to embodiment) were adapted from Ehrsson et al. (2008) and Longo et al. (2008) (see Supplementary Table S1) (Ehrsson et al., 2004; Longo et al., 2008), similar to modifications used by prior authors for closed loop prosthetic control (Marasco et al., 2011; Graczyk et al., 2018; Page et al., 2018; Valle et al., 2018). The control statements were included to assess suggestibility and the embodiment statements were included to assess perception of key components of embodiment which are location, ownership, and agency. Additional questions on agency and “loss of hand” were included as potential components of the RHI, modified from Longo et al. (2008). Four versions of this questionnaire with a randomized order of the questions were developed, and a randomly selected version of the questionnaire was administered after each condition. The participant was asked to rate the strength of their agreement or disagreement for each question by pointing on the Visual Analog Scale with their left index finger. The scale was graded from 0 mm (strongly disagree) to 100 mm (strongly agree), and the distance was measured in millimeters. Higher grades on the embodiment questions indicated a greater sense of embodiment.

### Statistical Analysis

Normality was assessed using Levene’s test. A paired sampled t-test was conducted to assess if there was a difference between the means of embodiment questions (Q1–Q5) and control questions (Q6–Q10) for each condition, to determine suggestibility.

A repeated measures Analysis of Variance (ANOVA) with Bonferroni correction was used on the average score of the embodiment questions. The factors for the ANOVA were Feedback Type and Feedback Condition, and levels of the factors were Brushing/Tapping and Synchronous/Asynchronous, respectively. The *F*-test of significance was used to assess the effects of the different independent variables. If significance was found, pairwise comparisons were made to assess where the differences lie. An α of 0.05 was used for all comparisons, and Bonferroni correction for multiple comparisons was utilized.

If a significant difference was detected between conditions on the average embodiment scores, then repeated measures ANOVA was run on the VAS response to each embodiment question (Q1–5) across conditions to determine if we could further detect differences among the responses to individual questions.

## Results

### Passive Prosthesis Embodiment

Responses to the questionnaire show that there was a statistically significant difference between the responses to embodiment items (Q1–Q5) and control items (Q6–Q10) for synchronous brushing feedback condition, as determined by paired sample *t*-test [*t*(20) = 5.1, *p* < 0.001]. Conversely, there was no statistically significant difference between participant’s responses to embodiment items and control items for the asynchronous brushing feedback condition; paired sample *t*-test [*t*(20) = 1.8, *p* = 0.095; Supplementary Table S2]. Both of these findings confirm that participants were not suggestible.

**FIGURE 3:**
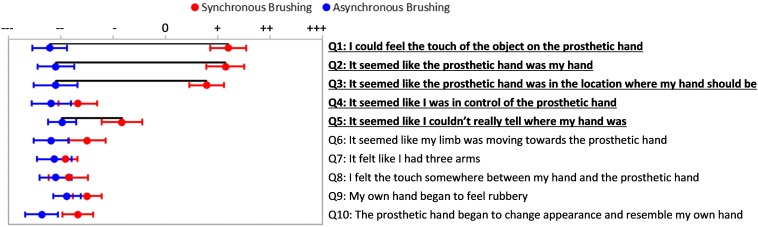
Passive Prosthesis Test: Questionnaire results for synchronous and asynchronous brushing. The questionnaire included these 10 statements, presented randomly. Statements 1–5 were used to describe aspects of the embodiment phenomena (Longo et al., 2008). Subjects indicated their response on a visual analog scale ranging from strongly disagree to strongly agree. Red points indicate mean responses for synchronous brushing condition and blue points indicate mean responses for asynchronous brushing condition. Bars extending from these points indicate standard error of the mean (SEM) response. Horizontal black lines indicate statistically significant tendency to evoke affirmative responses (*p* < 0.05).

Participants’ responses to embodiment questions (Q1–Q5) were statistically significantly different between all tested conditions as determined by repeated measures ANOVA [*F*(3, 60) = 9.8, *p* < 0.001]; *post hoc* analysis indicated all comparisons were significantly different, except for SB vs. ST (Supplementary Figure S1 and Supplementary Table S3). Further analysis of the responses to individual questions indicated that providing users with synchronous brushing feedback prompted a significantly higher sense of embodiment on 4 out 5 of the embodiment questions than when providing users with asynchronous brushing feedback (Q 1, 2, 3, and 5, Bonferroni *post hoc* test, *p* < 0.05). The only question that did not evoke a significantly stronger response with synchronous brushing was the agency question (Q4) (Figure 3).

**FIGURE 4:**
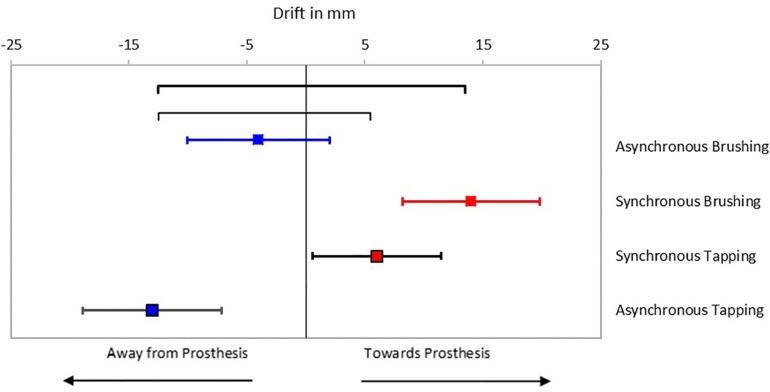
Passive Prosthesis Test: Mean proprioceptive drifts toward the prosthetic hand for each of the conditions. Error bars indicate standard error of the mean. Black horizontal lines indicate statistical significance at *p* < 0.05. Red blocks indicate synchronous feedback, and blue blocks indicate asynchronous feedback.

Results from the proprioceptive drift test showed a significant difference between testing conditions as determined by repeated measures ANOVA [*F*(3, 60) = 3.02, *p* = 0.036; Supplementary Table S3]. Bonferroni *post hoc* analysis showed that participants had statistically significantly higher drift toward the prosthesis after receiving synchronous brushing feedback (14 ± 5.8 mm) or synchronous tapping feedback (6 ± 5.5 mm) compared to asynchronous tapping feedback (-13 ± 5.9 mm) (*p* = 0.014 and *p* = 0.018, respectively; Figure 4). Temporal manipulation of the brushing feedback (AB) did not result in a statistically significant difference in proprioceptive drift compared to the synchronous conditions, although the trend was to drift away from the prosthesis (−4 ± 6.0 mm).

**FIGURE 5:**
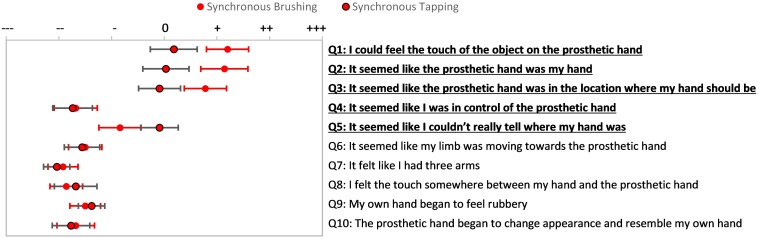
Passive Prosthesis Test: Questionnaire results for synchronous feedback conditions. Participants’ responses on statements 1–4 for synchronous brushing were higher than their corresponding responses for synchronous tapping. No statistically significant difference was found between both feedback types. Red points indicate mean responses for synchronous brushing condition and red with black outline dots indicate mean responses for synchronous tapping condition. Bars extending from these points indicate standard error of the mean (SEM) response.

To ensure that the mechanotactile system described in this work would have similar positive effects on prosthesis embodiment to brushing feedback, we also compared individual questionnaire responses for mechanotactile ST feedback to SB feedback (Figure 5). There was no statistically significant difference in responses to the embodiment statements between synchronous brushing and synchronous tapping conditions as determined by paired sample *t*-test [*t*(4) = 2.78, *p* = 0.26]. A Pearson product-moment correlation was performed to determine the relationship between responses to the questionnaire after receiving SB feedback and after receiving ST feedback in the passive prosthesis test. There was a strong, positive correlation between SB and ST, which was statistically significant (*r* = 0.919, *n* = 10, *p* = 0.00017).

It is worth noting that, although not statistically significant, brushing feedback tended to evoke more positive responses on the first three embodiment statements than the tapping feedback (Figure 5). Similarly, participants had a trend toward greater proprioceptive drift toward the prosthetic hand when provided with synchronous brushing feedback than when provided with synchronous tapping feedback (see Figure 4), however this was not statistically significant.

**FIGURE 6:**
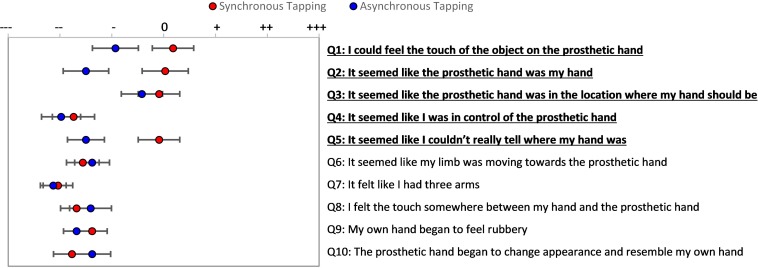
Passive Prosthesis Test: Questionnaire results for synchronous and asynchronous tapping feedback conditions. Subjects indicated their response on a visual analog scale ranging from strongly disagree to strongly agree. Red with black outline points indicate mean responses for synchronous tapping condition and blue with black outline points indicate mean responses for asynchronous tapping condition. Bars extending from these points indicate standard error of the mean (SEM) response.

Similar to the brushing condition, comparing the embodiment items to the control items for each tapping condition confirmed that participants were not suggestible, with a significant difference for the synchronous tapping condition [*t*(20) = 3.8, *p* = 0.001], but not for asynchronous tapping [*t*(20) = 2.0, *p* = 0.06]. The average of the embodiment question scores were statistically significantly different between synchronous and asynchronous conditions of the tapping feedback on Bonferroni *post hoc* analysis (*p* = 0.02; Supplementary Table S3). When examining individual questions, there was a trend for the temporal delay of the mechanotactile tapping feedback (AT) to negatively affect participants’ responses to embodiment statements in the questionnaire (Figure 6), although no statistically significant differences were found in responses to individual questions (*p* > 0.05).

### Active Prosthesis Embodiment

Having determined that tapping feedback using mechanotactile tactors promotes the embodiment of the prosthesis in a passive condition similar to brushing with the hand and forearm constrained in the brace, we next compared participants’ responses after actively controlling the prosthetic device with synchronous mechanotactile tapping feedback, delayed tapping feedback, and without tapping feedback. Responses to the questionnaire show that there was a statistically significant difference between the responses to embodiment items (Q1–Q5) and control items (Q6–Q10) within each condition, as determined by paired sample *t*-test [ST: *t*(18) = 5.5, *p* < 0.001; AT: *t*(18) = 4.0, *p* = 0.001; no feedback: *t*(18) = 2.6, *p* = 0.02; Supplementary Table S4].

**FIGURE 7:**
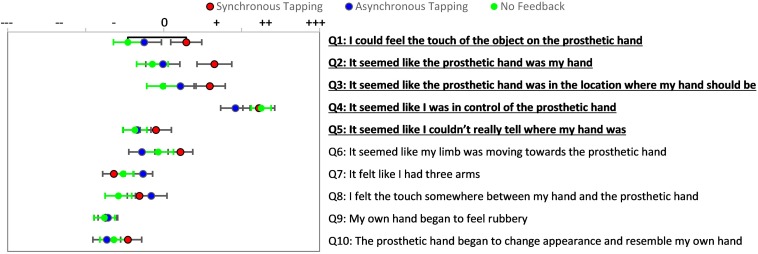
Active Prosthesis Test: Questionnaire results for controlling a prosthesis while receiving three feedback conditions. Participants’ responses on statements 1–5 for synchronous tapping were higher than responses for no feedback condition. Red with black outline points indicate mean responses for synchronous tapping condition, blue with black outline dots indicate mean responses for asynchronous tapping condition, and green dots indicate mean responses for no feedback condition. Bars extending from these points indicate standard error of the mean (SEM) response. Horizontal black line indicates statistically significant tendency to evoke affirmative responses (*p* < 0.05).

There was a significant difference in embodiment responses during the active prosthesis test with synchronous mechanotactile tapping feedback, delayed feedback, and no feedback as determined by repeated measures ANOVA [*F*(2, 36) = 7.2, *p* = 0.002]; Supplementary Figure S2 and Supplementary Table S5. Bonferroni *Post hoc* analysis showed that providing synchronous mechanotactile tapping feedback to participants while controlling the simulated prosthesis promoted statistically significant higher average responses to the embodiment questions than either asynchronous (*p* = 0.003) or no feedback (*p* = 0.003). When examining responses to individual embodiment questions, there was a significantly higher response to embodiment statement 1 with synchronous feedback compared to the response to the same statement when provided with no feedback (*p* = 0.004) (Figure 7). In contrast to the passive prosthesis experiment, high responses were seen on the agency question (Q4) for both the synchronous and no feedback conditions, with asynchronous tapping showing a non-significant trend of negatively affecting agency.

**FIGURE 8:**
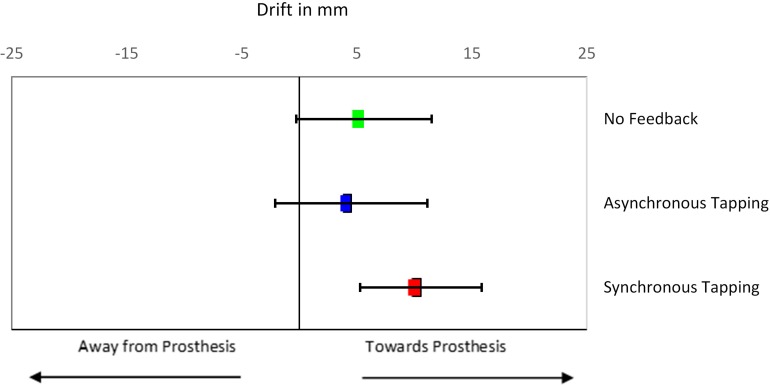
Active Prosthesis Test: Mean proprioceptive drifts toward the prosthetic hand after actively controlling it while receiving three types of feedback. Error bars indicate standard error of the mean. Red with black outline bar indicates synchronous tapping feedback, blue with black outline bar indicates asynchronous tapping feedback, and green bar indicates no feedback condition.

Results from the proprioceptive drift task in the Active Prosthesis Test showed all conditions resulted in some shift toward the prosthetic hand with a trend to higher proprioceptive drift for the synchronous tapping condition (shown in Figure 8), although not statistically significant (Supplementary Table S5).

## Discussion

Simulated upper-limb prosthesis systems are commonly used as an approximation to prostheses used by persons with upper-limb amputation, as a method of allowing able-bodied participants to actively control a prosthetic hand in a situation more similar to actual prosthesis use. Researchers have used various versions of simulated prostheses to investigate the performance of commercial prosthetic hands (Kyberd, 2011), performance of novel control strategies (Johansen et al., 2016; Shehata et al., 2018a), kinematic movement trajectories when using prosthetic hands (Williams et al., 2019), and, recently, the effect of providing sensory feedback to users on performance in functional tasks (Wilson et al., 2017; Engels et al., 2019). In this work, we used a sensorized simulated prosthesis to investigate the contribution of sensory feedback to the embodiment phenomenon during active motor control of the prosthesis, utilizing a common methodology in the literature, namely the RHI (Longo et al., 2008).

### Passive RHI

Wearing a simulated prosthesis in a passive situation evoked similar embodiment responses to prior work with the RHI. Specifically, we found that similar to other studies (Botvinick and Cohen, 1998; Tsakiris et al., 2005), participants tended to embody the prosthesis in the passive synchronous brushing condition as indicated by their positive responses to location and ownership statements on the questionnaire (particularly Q1–Q3) and high proprioceptive drift toward the device (Tsakiris et al., 2005). This finding was important, as the tactile contact of the brace on the skin of the intact forearm and hand may have presented a distracting stimulus that could alter the embodiment experience (Parmentier et al., 2011). However, brushing stimulation is not common in prosthesis user applications. In previous work (Marasco et al., 2011; Hebert et al., 2014), researchers relayed tactile feedback to persons with upper-limb loss by placing tactors on reinnervated skin areas; therefore, returning physiologically appropriate touch and pressure feedback from the prosthesis to the user through skin indentation. Implantable peripheral nerve interface approaches also most commonly report and utilize touch and pressure feedback corresponding to the digits (Wendelken et al., 2017; Schiefer et al., 2018; George et al., 2019). We, therefore, investigated the effect of using a mechanotactile stimulation of touch and pressure to provide the sensory information (Schoepp et al., 2018), to investigate its effect on simulated prosthesis embodiment. We found that synchronous touch and pressure stimulation evoked similar embodiment responses as brushing (no significant differences and strong positive correlation), although responses were blunted, consistent with prior literature (D’Alonzo and Cipriani, 2012). This finding was not surprising given that stroking a brush is known to evoke higher emotional affective responses (Crucianelli et al., 2013), and affect has also been considered an influential component of embodiment (Longo et al., 2008). The proprioceptive drift results confirmed that the synchronous stimulation conditions evoked higher displacement toward the prosthetic hand compared to the asynchronous tapping condition.

Similar to work in participants with amputation (Ehrsson et al., 2008; Marasco et al., 2011; Schmalzl et al., 2014), in our study, ownership and location aspects of embodiment were affected by the synchronicity of the feedback in the passive condition, however, the agency question was not affected by either synchronous or asynchronous brushing and tapping. This would be expected since participants did not have any control over the device during the first testing phase and therefore did not develop any sense of agency over the device. Our results for the passive (no voluntary control) test, therefore, showed that wearing a sensorized simulated prosthesis with integrated mechanotactile feedback can drive the perceptual shift of certain aspects of embodiment, namely ownership and location.

### Active Task

For the active prosthesis experiments, we found that active motor control induced a form of agency, reflected in the agency question and the proprioceptive drift, even for the no feedback condition. These results were consistent with prior findings in able-bodied subjects (Dummer et al., 2009) and those with limb amputation (Ehrsson et al., 2008; Sato et al., 2018). Having control over visualized movements of a robotic hand has been theorized to allow embodiment due to implicit knowledge of a kinesthetic sense, which contributes to making the experience personal (Dummer et al., 2009). The prosthesis user study by Marasco et al. (2018) examined the effect of restoring the kinesthetic sense of hand movement during an active grasping task on embodiment of a prosthetic hand. Results showed that providing kinesthetic feedback conferred a significantly greater sense of agency, but did not affect statements of limb ownership. Our findings support that the sense of agency can be induced by the use of the inherent kinesthetic sense associated with muscle contraction matched to active robotic control in our intact able-bodied participants (Kalckert and Ehrsson, 2012; Marotta et al., 2017), consistent with the restored kinesthesia and sense of agency in those with limb amputation (Marasco et al., 2018).

Adding synchronous tactile feedback to the active task enhanced embodiment responses compared to asynchronous or no feedback, when the average score of all embodiment questions were considered. Examining responses to individual questions revealed that synchronous tapping tended to result in the highest embodiment responses particularly for the first three questions, with no feedback resulting in the least amount of embodiment (see Figure 7). These findings are consistent with Graczyk et al. (2018), who reported positive responses to embodiment questions in participants with implantable neural interfaces after a sensory-enabled take-home trial; although, this embodiment did persist to the subsequent non-sensory enabled trial. Page et al. (2018) also found that providing sensory feedback to their participants with a neural interface in a passive condition significantly induced embodiment compared to the motor control only condition, however, there was no additional advantage of closed-loop control in enhancing the embodiment response. However, these studies did not conduct a deeper investigation of the sense of agency, which may, in fact, potentiate embodiment (Sato et al., 2018).

In our active prosthesis control experiment, although the addition of synchronous feedback added to specific aspects of embodiment, i.e., ownership and location (as represented by the first three questions), it did not affect agency. Agency was high with active control and not changed with adding synchronous feedback; however, asynchronous feedback tended to result in the lowest response to the agency question. This possible influence of asynchronous tapping raises an intriguing possibility for a potential mechanism to separate agency from ownership. Given that this finding was based on a single question, additional and more sensitive measures of agency would be helpful to test this hypothesis in future studies.

In the active prosthesis test, in addition to the high positive responses to the sense of agency statement on the questionnaire, participants demonstrated proprioceptive drift toward the simulated prosthesis for all conditions. This finding was unexpected as we hypothesized asynchronous tapping would cause a drift away from the prosthesis, as evidenced in the passive experiment. A possible influencing factor within our set up was that the asynchronous stimulation was provided at a fixed time delay, and the participant may have learned to incorporate that feedback, even though delayed (Blustein et al., 2018). Exploring the effect of timing of delayed feedback on feedback incorporation and real-time control may be an important area of future study.

Others have also noted a discrepancy between drift and subjective ownership responses in passive conditions of synchronous and asynchronous stroking (Rohde et al., 2011). In our work, participants were controlling a simulated prosthesis that was attached to their forearms to grasp and move objects. We propose that our participants utilized the kinesthetic sense of contracting their muscles to control the prosthesis to achieve the sense of agency over hand grasp. The proprioceptive senses associated with more proximal intact sensory organs around the shoulder and elbow could have affected the observed proprioceptive drift (Proske and Gandevia, 2012). There is also evidence that proprioceptive drift and agency may respond similarly (Tajima et al., 2015) and be task dependent (Shibuya et al., 2017). Proprioceptive drift may, therefore, be expected to differ between passive and active conditions, such that there is a stronger influence of motor control on this measurement of embodiment specifically.

### Limitations

There are limited opportunities to access participants with bidirectional sensory feedback systems and further limitations in manipulating sensory experiences to explore the impact of feedback type. We, therefore, chose to use able-bodied participants using a simulated prosthesis to determine if their embodiment responses would be similar to those with limb amputation, and potentially modifiable. However, it must be kept in mind that the inherent neurophysiological structures have not been interrupted as in those with limb amputation (Knecht et al., 1996), therefore, these participants are a proxy at best. These preliminary results suggest that the use of a sensory simulated prosthesis can induce embodiment responses (ownership and location) and may separately affect the construct of agency, even with the limited subjective measures employed. This approach opens an avenue for more in-depth exploration of this phenomenon that may then be applied to the sample of individuals with sensory-enabled upper limb prosthesis systems.

We used three traditional embodiment statements commonly cited in the literature, and included additional questions on agency and loss of own hand. The questions, originally derived from Botvinick and Cohen (Botvinick and Cohen, 1998) and modified by other authors (Ehrsson et al., 2008; Longo et al., 2008) were based on experiments designed for a passive experience rather than an active control situation, and may need to be further refined and validated for new experimental paradigms (Gonzalez-Franco and Peck, 2018). Other authors have similarly used analysis of three modified embodiment statements (Graczyk et al., 2018; Page et al., 2018; Valle et al., 2018), as well as answers to individual questions (Marasco et al., 2011; Valle et al., 2018) to interpret embodiment in close-loop prosthetic control conditions. A lack of multiple quantitative measures to assess embodiment and the related phenomenon (such as agency, location, and proprioception) is a clear limitation of this work. The use of subjective questionnaire ratings generally limits the interpretation of the findings and highlights the crucial lack of quantitative measures to address outstanding questions on the components of embodiment such as agency. More recent work on quantitative measures of agency and ownership, including intentional binding paradigms, incorporation measures, and internal model (Haggard et al., 2002; Moore and Obhi, 2012; Kühn et al., 2013; Blustein et al., 2018; Shehata et al., 2018b) may allow future work to more adequately parse out the contributions of sensory feedback and active motor control in an active prosthesis control situation.

## Conclusion

We have shown that a simulated prosthesis actively used for functional control activities may be embodied by able-bodied users (Ehrsson et al., 2008; Marasco et al., 2011; Schmalzl et al., 2014). In addition, we verified that mechanotactile sensory feedback might not only be useful for improving sense of ownership and location but also may have a modulating effect on the sense of agency when provided asynchronously during active motor control tasks. The simulated sensory-motor prosthesis system may allow us to manipulate the factors contributing to the complex phenomenon of embodiment, enabling future study of more precise quantitative measures of embodiment that do not rely as much on subjective perception, which will be crucial to advancing knowledge in this field.

## Data Availability Statement

The raw data supporting the conclusions of this article will be made available upon reasonable request, without undue reservation, to any qualified researcher.

## Ethics Statement

Written informed consent according to the University of Alberta Health Research Ethics Board was obtained from all participants before conducting the experiments (Pro00057340). This work is not a clinical trial. All experiments were conducted with able-bodied participants in a lab setting and not in a clinic.

## Author Contributions

JSH and ZEJ conceived the idea and designed the study. ZEJ conducted the experiments, summarized the data, and contributed to the manuscript draft. MR and AWS analyzed the data. AWS, MR, and JSH wrote and contributed to manuscript revisions. All authors approved the submitted version.

## Conflict of Interest

The authors declare that the research was conducted in the absence of any commercial or financial relationships that could be construed as a potential conflict of interest.

## Abbreviations

RHI: rubber hand illusion
SB: synchronous brushing
AB: asynchronous brushing
ST: synchronous tapping
AT: asynchronous tapping.
