# Supplementary material for: Mechanotactile Sensory Feedback Improves Embodiment of a Prosthetic Hand During Active Use
Source: Front Neurosci. 2020 Mar 26;14:263. doi: 10.3389/fnins.2020.00263 (PMC7113400; doi:10.3389/fnins.2020.00263)
Supplement: Supplementary file 1 [file Data_Sheet_1.PDF]

Appendix A – Questionnaire Items

Embodiment statements:

1) I could feel the touch of the object on the prosthetic hand
2) It seemed like the prosthetic hand was my hand
3) It seemed like the prosthetic hand was in the location where my hand should be
4) It seemed like I was in control of the prosthetic hand
5) It seemed like I couldn’t really tell where my hand was

Control Statements:

6) It seemed like my limb was moving towards the prosthetic hand
7) It felt like I had three arms
8) I felt the touch somewhere between my hand and the prosthetic hand
9) My own hand began to feel rubbery
10) The prosthetic hand began to change appearance and resemble my own hand

Table S1. Original embodiment statements from Longo et al., 2008 and Ehrsson et al., 2008 and associated modified embodiment statements for the experimentation. The word “rubber hand” was replaced with “prosthetic hand” and “brush” with “object” in order to apply to the active prosthesis test.

**Source**	**Statement**	**Modified Statement**
Ehrsson etal. 2008 (Q1)	I could feel the touch of the *brush* on the prosthetic hand	Q1. I felt the touch of the *object* on the prosthetic hand
Longo et al. 2008 (Q4)<br><br>Ehrsson etal. 2008 (Q3)	It seemed like the *rubber hand* was my hand.<br>It felt as if the prosthetic hand was my hand	Q2. It seemed like the *prosthetic* hand was my hand.
Longo et al. 2008 (Q7)	It seemed like the *rubber hand* was in the location where my hand was	Q3. It seemed like the *prosthetic hand* was in the location where my hand should be
Longo et al. 2008 (Q10)	It seemed like I was in control of the rubber hand	Q4. It seemed like I was in control of the prosthetic hand
Longo et al. 2008 (Q14)	It seemed like I couldn’t really tell where my hand was	Q5. It seemed like I couldn’t really tell where my hand was

Appendix B – Results of Statistical Analysis

Table S2. Paired sample t-test for Passive Prosthesis Test embodiment vs. control questions

Measurable variable	***Groups***	***df***	***T Stat***	***P value***	***t-two tailed***
**SB VAS score**	Embodiment Q vs. Control Q	20	5.08	0.00006	2.086
**AB VAS score**	Embodiment Q vs. Control Q	20	1.75	0.095	2.086
**ST VAS score**	Embodiment Q vs. Control Q	20	3.78	0.0012	2.086
**AT VAS score**	Embodiment Q vs. Control Q	20	2.035	0.055	2.086

Table S3. Repeated measures ANOVA for Passive Prosthesis Test

Measurable variable	***Groups***	***df***	***F***	***P value***	***F crit***
Embodiment questions (Q1-Q5) average score	SB, ST, AB, and AT	3,60	9.804613	0.0000235	2.758078
Bonferroni Post-hoc	SB vs. AB			0.0000319	
	SB vs. ST			0.173	
	SB vs. AT			0.0077	
	AB vs. ST			0.0045	
	AB vs. AT			0.0442	
	ST vs. AT			0.02	
Proprioceptive Drift score	SB, ST, AB, and AT	3,60	3.023137	0.036483	2.758078
Bonferroni Post-hoc	SB vs. AB			0.193	
	SB vs. ST			0.405	
	SB vs. AT			0.014	
	AB vs. ST			0.5	
	AB vs. AT			0.192	
	ST vs. AT			0.018	

Table S4. Paired sample t-test for Active Prosthesis Test embodiment vs. control questions

Measurable variable	***Groups***	***df***	***T Stat***	***P value***	***t-two tailed***
**ST VAS score**	Embodiment Q vs. Control Q	18	5.55	0.000029	2.1
**AT VAS score**	Embodiment Q vs. Control Q	18	3.96	0.0009	2.1
**NIL VAS score**	Embodiment Q vs. Control Q	18	2.6	0.018	2.1

Table S5. Repeated measures ANOVA for Active prosthesis test

Measurable variable	***Groups***	***df***	***F***	***P value***	***F crit***
Embodiment questions (Q1-Q5) average score	ST, AT, and NIL	2,36	7.1754	0.00238	3.26
Bonferroni Post-hoc	ST vs. AT			0.0027	
	ST vs. NIL			0.00282	
	AT vs. NIL			0.526	
Proprioceptive Drift score	ST, AT, and NIL	2,36	0.25	0.781	3.26
Bonferroni Post-hoc	ST vs. AT			0.48	
	ST vs. NIL			0.612	
	AT vs. NIL			0.91	

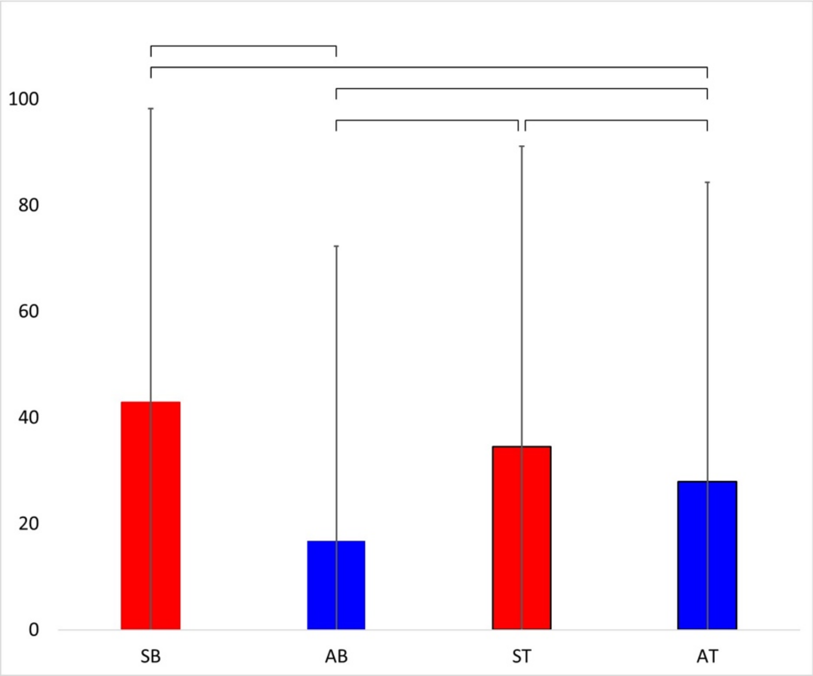

Figure S1. Passive Prosthesis Test: Average of participant responses to embodiment questions (Q1–Q5), compared across conditions. All comparisons were significantly different, other than SB to ST condition (as reported in Table S3).

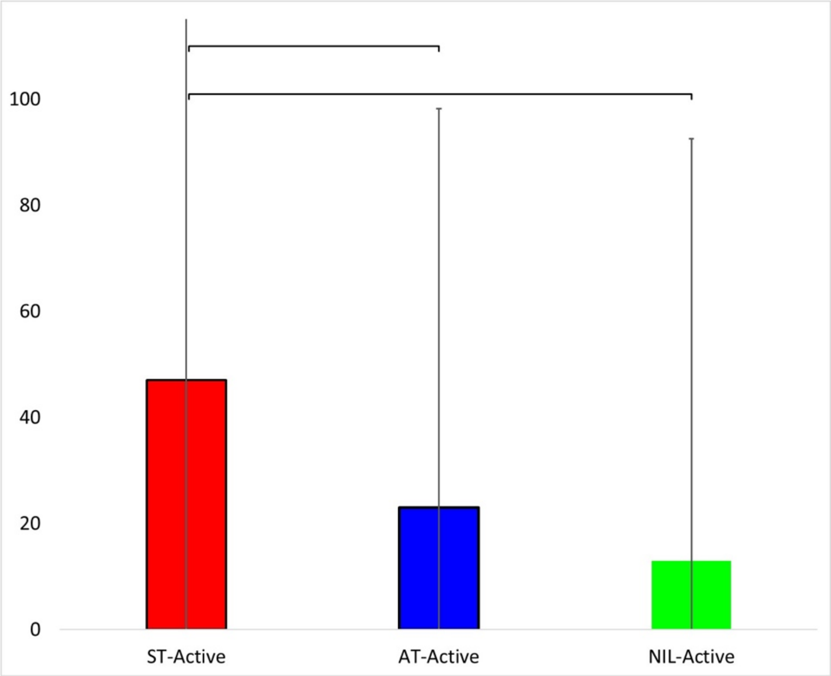

Figure S2. Active Prosthesis Test: Average of participant responses to embodiment questions (Q1–Q5), compared across conditions. ST was significantly different to At and to Nil (as reported in Table S5).
